# Palmitic acid–induced microRNA-143-5p expression promotes the epithelial–mesenchymal transition of retinal pigment epithelium via negatively regulating JDP2 

**DOI:** 10.18632/aging.204684

**Published:** 2023-04-25

**Authors:** Yunlin Tian, Juan Shao, Shuwei Bai, Zhiguo Xu, Chunchao Bi

**Affiliations:** 1 Department of Ophthalmology, Shaanxi Eye Hospital, Xi’an People’s Hospital (Xi'an Fourth Hospital), Affiliated Guangren Hospital, School of Medicine, Xi’an Jiaotong University, Xi’an 710004, China

**Keywords:** epithelial–mesenchymal transition (EMT), retinal pigment epithelial (RPE) cells, miR-143-5p, JDP2

## Abstract

Background: The epithelial–mesenchymal transition (EMT) of retinal pigment epithelial (RPE) cells is the most crucial step in the etiopathogenesis of proliferative vitreoretinopathy. This study aimed to investigate the role of miR-143-5p in the EMT of RPE cells induced by palmitic acid (PA).

Methods: ARPE-19 cells were treated with PA to induce EMT, followed by E-cadherin and α-smooth muscle actin (α-SMA) expression and the microRNA expression profile analyses. Subsequently, miR-143-5p mimics/inhibitors, and plasmids expressing its predicted target gene c-JUN-dimerization protein 2 (*JDP2*), were transfected in ARPE-19 cells using lipofectamine 3000, and followed by PA treatment. Their impacts on EMT were explored using wound healing and Western blot assays. Additionally, miR-143-5p mimics and JDP2-expressing plasmid were co-transfected into ARPE-19 cells and treated with PA to explore whether PA induced EMT of ARPE-19 cells via the miR-143-5p/JDP2 axis.

Results: PA decreased E-cadherin expression and increased those of α-SMA and miR-143-5p. Inhibiting miR-143-5p suppressed the migration of ARPE-19 cells and altered the expressions of E-cadherin and α-SMA. However, additional PA treatment attenuated these alterations. *JDP2* was a target of miR-143-5p. Overexpression of JDP2 inhibited the EMT of ARPE-19 cells, resulting in α-SMA downregulation and E-cadherin upregulation, which were reversed by additional PA treatment via inhibiting JDP2 expression. Overexpression of miR-143-5p reversed the effect of JDP2 on the EMT of ARPE-19 cells and additional PA treatment markedly enhanced the effect of miR-143-5p mimics.

Conclusion: PA promotes EMT of ARPE-19 cells via regulating the miR-143-5p/JDP2 axis, and these findings provide significant insights into the potential targeting of this axis to treat proliferative vitreoretinopathy.

## INTRODUCTION

Proliferative vitreoretinopathy (PVR) is the abnormal process of vision damage due to scarring that occurs as a complication after treatment for rhegmatogenous retinal detachment [1–3]. The incidence of PVR after surgery is approximately 5%–10% and is also the major cause of surgical failure [4]. Retinal pigment epithelial (RPE) cells are highly polarized and terminally differentiated cells that perform various biological functions, including serving as ion channels, absorbing scattered light, and providing barrier activity [5]. RPE cells play a major role in PVR. They can induce PVR initiation and development by migrating and dedifferentiating through a retinal break and proliferating on the retinal surface [6]. RPE cells are believed to promote the pathological process through epithelial–mesenchymal transition (EMT) and migration to the supraretinal region [7]. EMT is driven by Snail, zinc-finger E-box-binding (ZEB) and basic helix–loop–helix (bHLH) transcription factors that repress epithelial marker genes (such as E-cadherin) and activate genes associated with the mesenchymal phenotype (such as α-SMA, Fibronectin, and Vimentin) [8]. Several studies have shown that RPE cells are capable of undergoing EMT and migrating into the neuroretina, a process that is critical to the pathogenesis of PVR [9, 10]. Many animal studies on PVR have also demonstrated that EMT occurs in RPE cells, once PVR is induced [11–13].

Palmitic acid (PA) is the most ordinary saturated long-chain fatty acid in animals and plants that can cause cardiovascular diseases and cancers via various mechanisms, including the aberrant expression of microRNAs (miRNAs) [14, 15]. miRNAs are small noncoding RNAs with length of 17–25 nucleotides that play a pivotal role in the posttranscriptional regulation of mRNAs for translation, and they influence diverse biological activities [16, 17]. To date, many biological functions of miRNAs have not been completely elucidated. Emerging evidence shows that miRNAs are correlated with the EMT of RPE, and the dysregulation of miRNAs has been previously implicated in the pathogenesis of PVR [18]. Moreover, a recent study has suggested that miRNAs play a critical role in the EMT of RPE cells. For example, miR-204/211 plays a pivotal role in the differentiation of RPE cells [19]. Several studies have also confirmed the importance of miRNA-204/211 in preventing the EMT of RPE cells [19]. Furthermore, some RNA, including miR-29b, miR-124, miR-20, miR-21, miR-182, and miR-204/211, have been verified as miRNAs with some functions in PVR [20–22]. These studies suggest that in the development of PVR, miRNAs are molecular regulatory or potential therapeutic targets.

miRNA-143 is one of the best-characterized miRNAs located at a fragile site of chromosome 5: 149428918-149429023 (+) (https://www.mirbase.org/cgi-bin/mirna_entry.pl?acc=MIMAT0004599). Many previously mentioned studies have shown that miR-143 functions as a tumor suppressor in the development of several cancers, including colorectal cancer, osteosarcoma, ovarian cancer, and gastric cancer [22–25]. However, the role of miR-143 in the EMT regulation of non-cancerous diseases remains rarely reported. miR-143 has two duplexes: miR-143-3p, the guide strand and miR-143-5p, the passenger strand [26]. A recent study demonstrated that miR-143-5p was significantly upregulated in cystic fibrosis, the progression of which is widely accompanied by EMT [27], suggesting that miR-143-5p might play critical role in EMT or fibrosis associated non-cancerous diseases. However, the role of miR-143-5p in EMT of PVR remains unclear. C-JUN dimerization protein 2 (JDP2) is a transcriptional repressor of follicle-stimulating hormone β (FSH β) and has diverse biological functions in different cells, such as the promotion of apoptosis and migration [28]. Bioinformatics analysis predicted that JDP2 mRNA possesses the binding site of miR-143-5p. However, the functions of miR-143-5p and JDP2 and their involvements in the EMT of RPE cells remain unclear.

Hence, in the present study, we sought to reveal how miR-143-5p functions in the EMT of RPE cells. We showed that PA induced the upregulation of miR-143-5p expression in a human retinal pigment epithelial cell line ARPE-19 and that miR-143-5p mimics promoted EMT of ARPE-19 cells. Additionally, we conducted further mechanistic investigations and identified c-JUN-dimerization protein 2 (*JDP2*) as a direct target of miR-143-5p. Aberrant JDP2 expression remarkably suppressed the EMT of ARPE-19 cells and reversed the effects of miR-143-5p overexpression in human ARPE-19 cells. Our data from *in vitro* experiments offer new insights into the molecular functions of miR-143-5p in human ARPE-19 cells and suggest that the miR-143-5p/JDP2 axis can be targeted to decrease the severity of PVR through inhibiting the EMT of RPE cells.

## MATERIALS AND METHODS

### Cell culture and treatments

Adult human ARPE-19 cell line was obtained from the American Type Cell Collection (ATCC^®^ CRL-2302™, Manassas, VA, USA). This cell line was guaranteed by the vendor as free of mycoplasma contamination. Cells were maintained in complete Dulbecco’s Modified Eagle Medium/Nutrient Mixture F-12 (DMEM/F12) supplemented with 10% fetal bovine serum (FBS; HyClone™, Thermo Fisher Scientific, Waltham, MA, USA), which consisted of 200 mM L-glutamine, 15 mM HEPES, 100 U/mL penicillin, and 100 μg/mL streptomycin (Gibco; Thermo Fisher Scientific). Cells were cultured in a moist atmosphere with 5% CO_2_ at 37°C. The culture medium was renewed two to three times weekly, and cells were subcultivated after trypsinization. PA (Sigma-Aldrich, St. Louis, MO, USA), was dissolved in DMSO (available from Sigma-Aldrich) and stored at 1 M concentration. ARPE-19 cells were induced with PA at the optimal concentrations for 48 h.

### Transfection of miRNA mimics and inhibitors

The miR-143-5p mimics, miR-143-5p inhibitors, and negative control (NC) mimics and inhibitors were chemically synthesized and prepared by GenePharma (Shanghai, China). ARPE-19 cells were plated in 24-well plates at a density of 5 × 10^4^/mL and cultured overnight once in the logarithmic growth phase. The optimal miR-143-5p mimics, miR-143-5p inhibitors, or NC mimics and inhibitors, were then introduced into ARPE-19 cells using Lipofectamine 3000 Transfection Reagent (Thermo Fisher Scientific) following the manufacturer’s instructions. After 48 h of transfection, cell samples were collected for further analysis.

### Construction of JDP2 overexpression plasmids

The DNA coding sequence for JDP2 was cloned into the pcDNA3.1 (+) expression vector (Thermo Fisher Scientific), and the empty vector pcDNA3.1 was used as NC. The ARPE-19 cells in the logarithmic growth phase were transfected with pcDNA3.1 (+) -JDP2 or pcDNA3.1 (+) using Lipofectamine 3000 Transfection Reagent (Thermo Fisher Scientific) following the manufacturer’s instructions. After 48 h of transfection, the cells were collected for further analysis.

### Wound healing assay

After transfection with pcDNA3.1-JDP2 or miR-143-5p mimics/inhibitors, ARPE-19 cells were seeded in six-well plates overnight with 80% confluency. Then, scratches were made on plates with a 200 μL pipette tip and cells were rinsed three times with PBS to remove suspension cells. Following this, cells were cultured in fresh medium for 48 h. Cells were photographed under an optical microscope at 0 and 48 h, and analyzed using the Image J software (NIH Image, Bethesda, MD, USA).

### Reverse transcription–quantitative PCR

According to the manufacturer’s protocols, the total RNAs and miRNAs of the ARPE-19 cells were extracted using Trizol (Labref, Xi’an, China) and the miRNeasy mini kit (Qiagen, Hilden, Germany), respectively. Reverse transcription (RT) was conducted using the PrimeScript^®^ RT Master Mix (Takara Bio Inc., Shiga Prefecture, Japan) for mRNA quantification and the miScript II RT kit with a universal tag (Qiagen) for miRNA quantification. Quantitative PCR (qPCR) was conducted using the standard protocol for the SYBR Green PCR kit on an AB7500 RT-PCR system (Thermo Fisher Scientific). The relative gene expression was normalized to the levels of GAPDH (for mRNA) or U6 (for miRNA) using the comparative threshold cycle (2^−ΔΔCT^) method. Supplementary Table 1 lists the primers used for the qRT-PCR analysis of JDP2, GAPDH, miRNA-143-5p, and U6. Each experiment was repeated three times, and each sample was run in triplicate.

### Dual-luciferase reporter assay

The mRNA candidates of miR-143-5p were predicted using three online tools: TargetScan [29] (http://www.targetscan.org/), miRDB [30] (http://mirdb.org/), and microT [31] (http://diana.imis.athena-innovation.gr/DianaTools/index.php). The predicted binding site within the 3′ untranslated region (UTR) of JDP2 and the corresponding mutated binding region were cloned into a pGL3-Basic vector. The vectors were co-transfected into ARPE-19 cells together with the miR-143-5p mimics or scramble control miR-NC. After 48 h of transfection, the cells were collected and subjected to luciferase activity analysis using a dual-luciferase assay kit (Promega) and a GloMax luminometer (Promega), following the manufacturer’s instructions. Renilla luciferase activity was measured and normalized to firefly luciferase activity, and the relative luciferase activity of the control group was calculated.

### Western blot assay

ARPE-19 cells were lysed with PIRA assay buffer (AccuRef, Xi’an, China) supplemented with inhibitors, such as proteinase and phosphatase inhibitors (Selleck, Houston, TX, USA). Equal amounts of total protein (30 μg) were loaded and separated by 10% sodium dodecyl sulfate–polyacrylamide gel electrophoresis and transferred to polyvinylidene fluoride (PVDF) membranes (EMD Millipore, Billerica, MA, USA). The PVDF membranes were cut into small pieces containing target protein bands as designated by a protein ladder (LabRef, Xi’an, China). The PVDF membranes were then blocked with 5% nonfat milk at 37°C for 1 h and were further immunoblotted with primary antibodies at 4°C overnight. After washing three times in PBS containing 0.1% Tween-20, the PVDF membranes were incubated with horseradish peroxidase–labeled secondary anti-rabbit or anti-mouse polyclonal antibodies for 1 hour at room temperature. Proteins of interest were visualized using an enhanced chemiluminescence kit (EMD Millipore), and band intensities were quantified by densitometry using ImageJ software (National Institutes of Health, Bethesda, MD, USA). Supplementary Table 2 provides more detailed information on the antibodies used for the western blot assays.

### Statistics

The data are expressed as the mean ± standard deviation (SD) of at least three separate experiments. The Shapiro-Wilk test was used to test the normality distribution of the dataset. Differences between the means were determined using Student’s *t* test or one-way analysis of variance, followed by Dunnett’s post hoc test for multiple comparisons. The differences were considered to be significant at ^*^P < 0.05 and ^**^P < 0.01. Statistical analyses were conducted using GraphPad Prism 5.0 software (GraphPad Software, San Diego, CA, USA).

### Data availability statement

The datasets used and/or analyzed during the current study are available from the corresponding author upon reasonable request.

## RESULTS

### PA increased miR-143-5p expression in ARPE-19 cells

**Figure 1:**
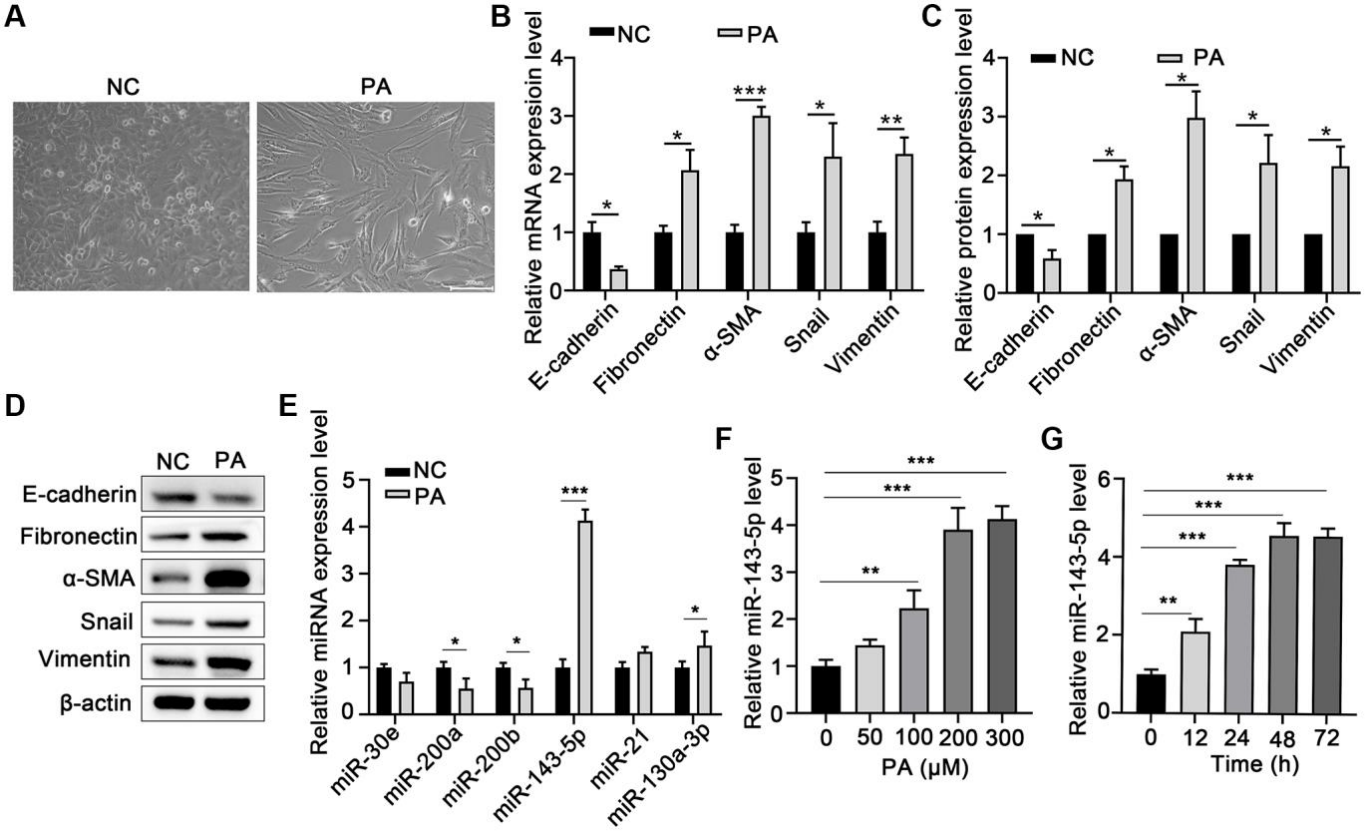
**PA induced EMT and increased miR-143-5p expression in ARPE-19 cells.** (**A**) Morphological alteration of ARPE-19 cells after treating with PA. Scale bar, 200 μm. (**B**) mRNA expression of E-cadherin, Fibronectin, α-SMA, Snail, and Vimentin as determined via quantitative PCR (qPCR) in ARPE-19 cells after PA treatment (*n* = 3 for each group). (**C**, **D**) The protein expression of E-cadherin, Fibronectin, α-SMA, Snail, and Vimentin determined using western blot in ARPE-19 cells after PA treatment (*n* = 3 for each group). (**E**) The expression of the indicated miRNAs in untreated control (NC) ARPE-19 cells, and ARPE-19 cells treated with PA (*n* = 3 for each group). (**F**) qPCR assays in dose-course experiments showed that the optimum PA concentration for inducing miR-143-5p expression in RPE cells was 200 μM (*n* = 5 for each group). (**G**) Results of time-course experiments demonstrated that the optimum duration of PA induction of miR-143-5p expression in RPE cells was 48 h (*n* = 5 for each group). Data are expressed as mean ± standard deviation; ^*^P < 0.05, ^**^P < 0.01, and ^***^P < 0.001, as compared with the indicated controls.

Morphological analysis revealed that PA obviously induced EMT in ARPE-19 cells (Figure 1A). To evaluate the effect of PA on the promotion of EMT in RPE cells, the mRNA and protein levels of E-cadherin or α-smooth muscle actin (α-SMA) after PA treatment of ARPE-19 cells were detected via qPCR and Western blot assays, respectively. PA treatment significantly decreased the expression of E-cadherin, but increased both mRNA and protein expression of Fibronectin, α-SMA, Snail, and Vimentin (Figure 1B–1D), suggesting that PA induced the EMT of ARPE-19 cells. To explore the potential association between miRNA and the PA-mediated EMT of ARPE cells, we compared the miRNA expression profile in ARPE-19 cells without and with PA treatment. As shown in Figure 1E, the relative expression of miR-143-5p increased by approximately four-fold, and was thus the most significantly altered miRNA among the pre-selected six candidate miRNAs according to our search of publications on EMT-related miRNAs. Further, ARPE-19 cells were treated with PA at different concentrations or at different durations to determine the optimal concentration and time of PA-induced EMT of the ARPE-19 cells. qPCR analyses revealed that the optimum concentration of PA and treatment duration for the induction of miR-143-5p expression in ARPE-19 cells was 200 μM (Figure 1F) at 48 h (Figure 1G). In addition, PA at a concentration no more than 300 μM did not decrease the viability of ARPE-19 cells (Supplementary Figure 1). Based on these findings, 200 μM PA treatment for 48 h was the treatment regimen used in subsequent analyses.

### PA-induced miR-143-5p expression promoted the EMT of RPE cells

**Figure 2:**
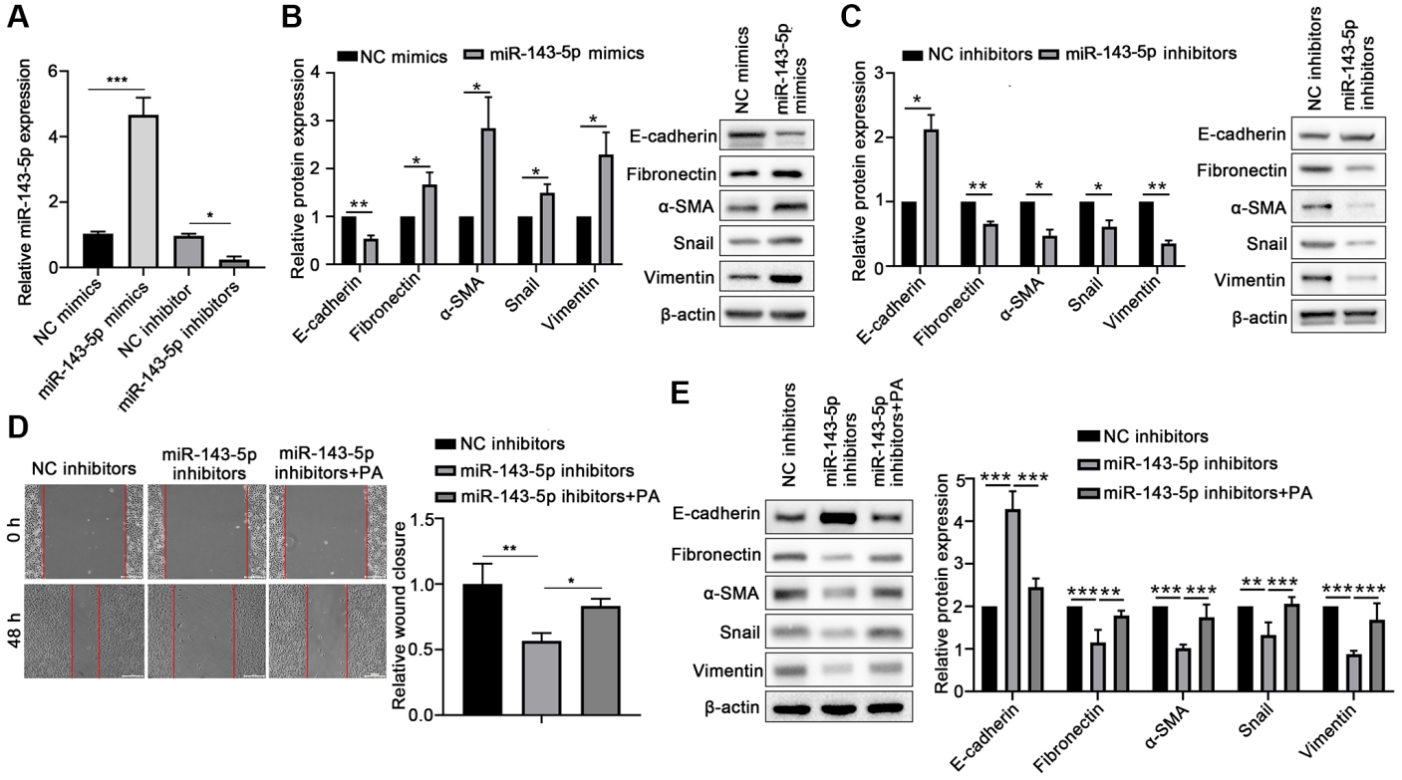
**PA enhanced the effect of miR-143-5p in promoting the EMT of ARPE-19 cells.** (**A**) miR-143-5p expression in ARPE-19 cells was determined via quantitative PCR; *n* = 3 for each group. (**B**) Protein expression of E-cadherin, Fibronectin, α-SMA, Snail, and Vimentin protein levels in ARPE-19 cells transfected with NC or miR-143-5p mimics (*n* = 3 for each group). (**C**) Protein expression of E-cadherin, Fibronectin, α-SMA, Snail, and Vimentin in ARPE-19 cells transfected with NC or miR-143-5p inhibitors (*n* = 3 for each group). (**D**) Migration of ARPE-19 cells after the overexpression of miR-143-5p inhibitors or/and PA treatment (*n* = 3 for each group). (**E**) Expression of E-cadherin, Fibronectin, α-SMA, Snail, and Vimentin in ARPE-19 cells after the overexpression of miR-143-5p inhibitors or/and PA treatment (*n* = 3 for each group). ^*^P < 0.05, ^**^P < 0.01, and ^***^P < 0.001, as compared with the indicated group.

To confirm the role of miR-143-5p in the EMT of ARPE-19 cells, the ARPE-19 cells were transfected with miRNA-143-5p mimics/inhibitors or their corresponding NC (Figure 2A), and the expression of associated markers was determined. Western blot analyses revealed that the protein level of E-cadherin significantly decreased, but the protein levels of Fibronectin, α-SMA, Snail, and Vimentin markedly increased in the miR-143-5p mimics group as compared with the NC group (Figure 2B). However, ARPE-19 cells transfected with miR-143-5p inhibitors showed significantly increased E-cadherin expression but decreased the Fibronectin, α-SMA, Snail, and Vimentin expression compared with the NC inhibitors group (Figure 2C). Furthermore, the wound healing assay showed that the overexpression of miR-143-5p inhibitor markedly decreased the migration of ARPE cells, whereas PA treatment reversed this effect (Figure 2D). Additionally, Western blot analysis also demonstrated that PA treatment obviously decreased the upregulation of E-cadherin by miR-143-5p inhibitors, but attenuated Fibronectin, α-SMA, Snail, and Vimentin expression (Figure 2E). All the above results indicate that PA-induced miR-143-5p distinctly promoted the EMT of ARPE cells.

### JDP2 was a direct target of miR-143-5p in human ARPE-19 cells

**Figure 3:**
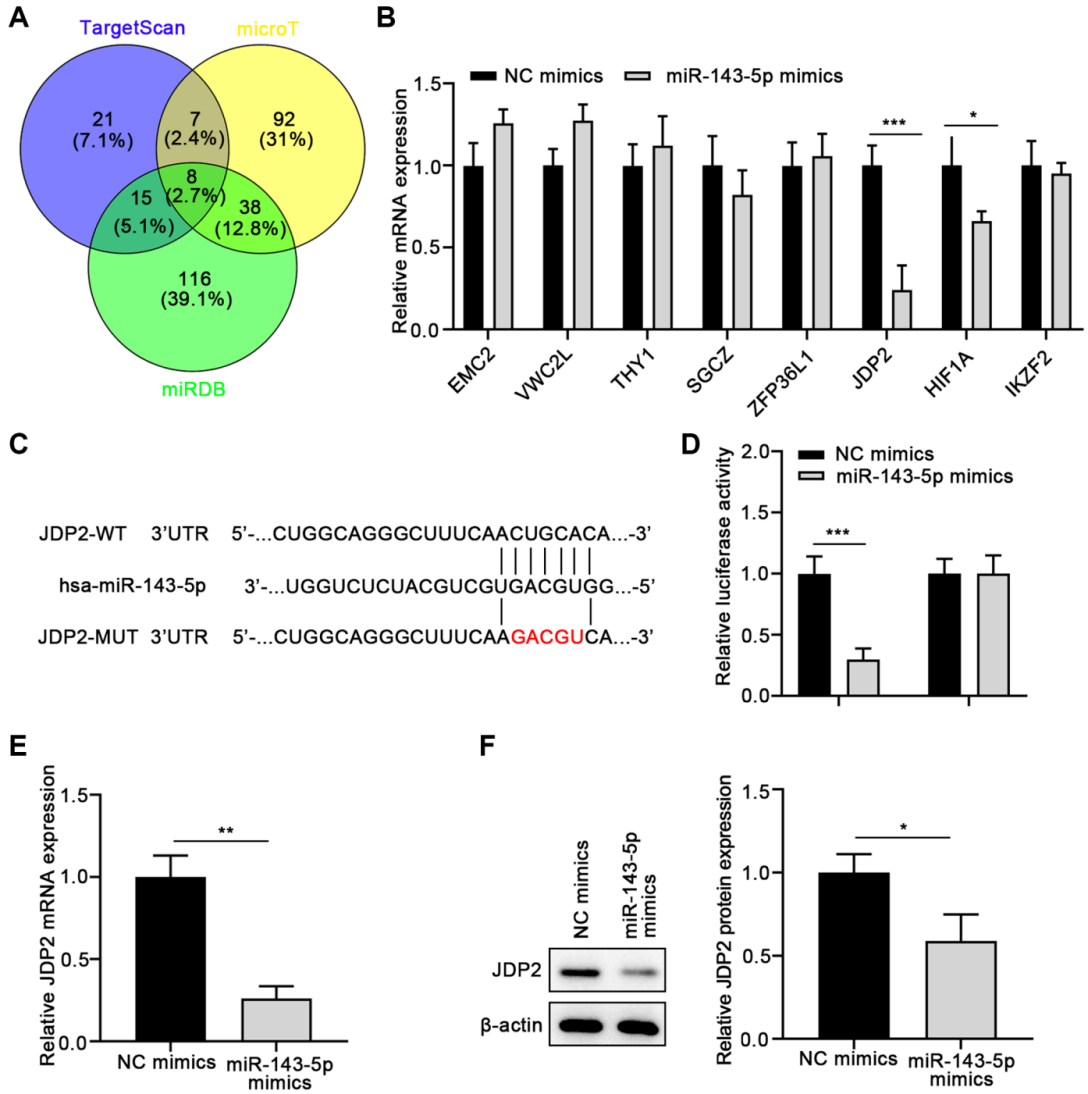
**JDP2 is a direct target of miR-143-5p in ARPE-19 cells.** (**A**) The underlying targets of miR-143-5p predicted using TargetScan, microT, and miRDB online tools followed by Venn analysis. (**B**) The qPCR assay showed that JDP2 mRNA expression was most significantly decreased in ARPE-19 cells after transfection of miR-143-5p mimics, compared with NC. (**C**) Diagrams showing the putative binding sites of miR-143-5p and the corresponding wild-type and mutant sites of JDP2 mRNA 3′ untranslated region (UTR). (**D**) miR-143-5p markedly suppressed the luciferase activity that carried the wild-type (WT) but not the mutant (MUT) 3′ UTR of JDP2. (**E**) The relative mRNA of JDP2 markedly decreased in ARPE-19 cells after transfection with miR-143-5p mimics (*n* = 3 for each group). (**F**) Relative JDP2 protein expression significantly in ARPE-19 cells after transfection with miR-143-5p mimics (*n* = 3 for each group). ^*^P < 0.05, ^**^P < 0.01, and ^***^P < 0.001, as compared with the indicated NC group.

To further investigate the molecular mechanisms underlying the promotion of miR-143-5p-mediated EMT of ARPE cells, the underlying target genes of miR-143-5p were predicted using the TargetScan, microT, and miRDB databases, followed by Venn analysis (Figure 3A). Eight overlapped genes were screen on the three databases. Therefore, the expressions of these eight genes were determined in ARPE cells after miR-143-5p mimics transfection. The results suggest that the overexpression of miR-143-5p mimics significantly decreased the expression of JDP2 and HIF1A, with JDP2 being the most significantly downregulated (Figure 3B). Thus, JDP2 was chosen as the target gene for the subsequent investigations. The consensus binding sites between miRNA-143-5p and JDP2 was presented in Figure 3C. The dual-luciferase assay was conducted to confirm whether JDP2 was a direct target of miRNA-143-5p in human ARPE-19 cells. Our data showed that the luciferase activities decreased notably after co-transfection of wild-type JDP2 3′UTR and miRNA-143-5p mimics. However, these activities remained largely unchanged after co-transfection of both miRNA-143-5p mimics and mutated JDP2 3′UTR, compared with the NC group (Figure 3D). These results indicate the direct interaction between miRNA-143-5p and 3′UTR of JDP2. Additionally, the JDP2 mRNA expression was significantly increased in human ARPE-19 cells transfected with miRNA-143-5p inhibitor, compared with the cells transfected with NC inhibitors (Figure 3E). Moreover, Western blot assays also demonstrated a marked increase in JDP2 protein expression in human ARPE-19 cells transfected with miRNA-143-5p inhibitor (Figure 3F). Therefore, the above results indicate that JDP2 is a direct downstream target of miRNA-143-5p in human ARPE-19 cells.

### JDP2 overexpression inhibited the EMT of ARPE-19 cells, which was attenuated by PA treatment

**Figure 4:**
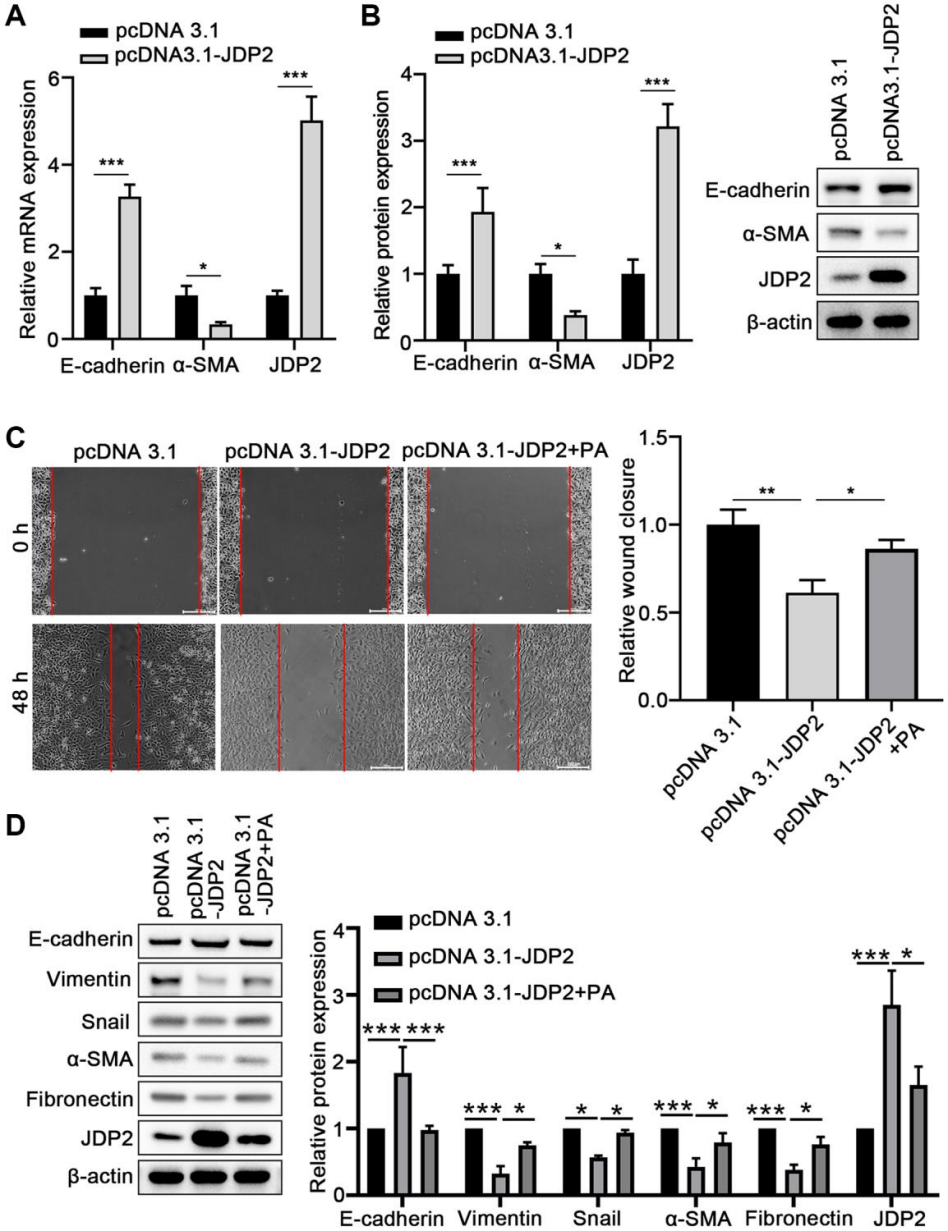
**JDP2 inhibited the EMT of ARPE-19 cells.** (**A**) The mRNA levels of E-cadherin and α-SMA in ARPE-19 cells transfected with empty vector or JDP2-expressing plasmid were determined via quantitative PCR. (**B**) Relative protein expression E-cadherin and α-SMA in ARPE-19 cells transfected with empty vector or JDP2-expressing plasmid (*n* = 3 for each group). (**C**) Migration of ARPE-19 cells after the overexpression of JDP2 or/and PA treatment (*n* = 3 for each group). (**D**) Expression of E-cadherin, Vimentin, Snail, α-SMA, Fibronectin, and JDP2 in ARPE-19 cells after the overexpression of JDP2 or/and PA treatment (*n* = 3 for each group). ^*^P < 0.05, ^**^P < 0.01, and ^***^P < 0.001, as compared with the indicated controls.

To further investigate the molecular mechanisms underlying the promotion of the EMT of ARPE-19 cells mediated by PA in ARPE-19 cells, JDP2 was overexpressed in ARPE-19 cells, and EMT was determined. The results suggest that the overexpression of JDP2 significantly increased the E-cadherin expression but decreased α-SMA expression at both mRNA and protein levels (Figure 4A, 4B). The wound healing assay showed that JDP2 overexpression significantly inhibited the migration of ARPE-19 cells, but PA treatment reversed this effect (Figure 4C). The Western blot assays also demonstrated that PA treatment reversed the effect of JDP2’s effects in increasing E-cadherin expression and decreasing those of Vimentin, Snail, α-SMA, and Fibronectin (Figure 4D). Taken together, these findings suggest that JDP2 inhibits the EMT of ARPE-19 cells, which can be attenuated by additional PA treatment.

### PA promoted the EMT of ARPE-19 cells via the miR-143-5p/JDP2 axis

**Figure 5:**
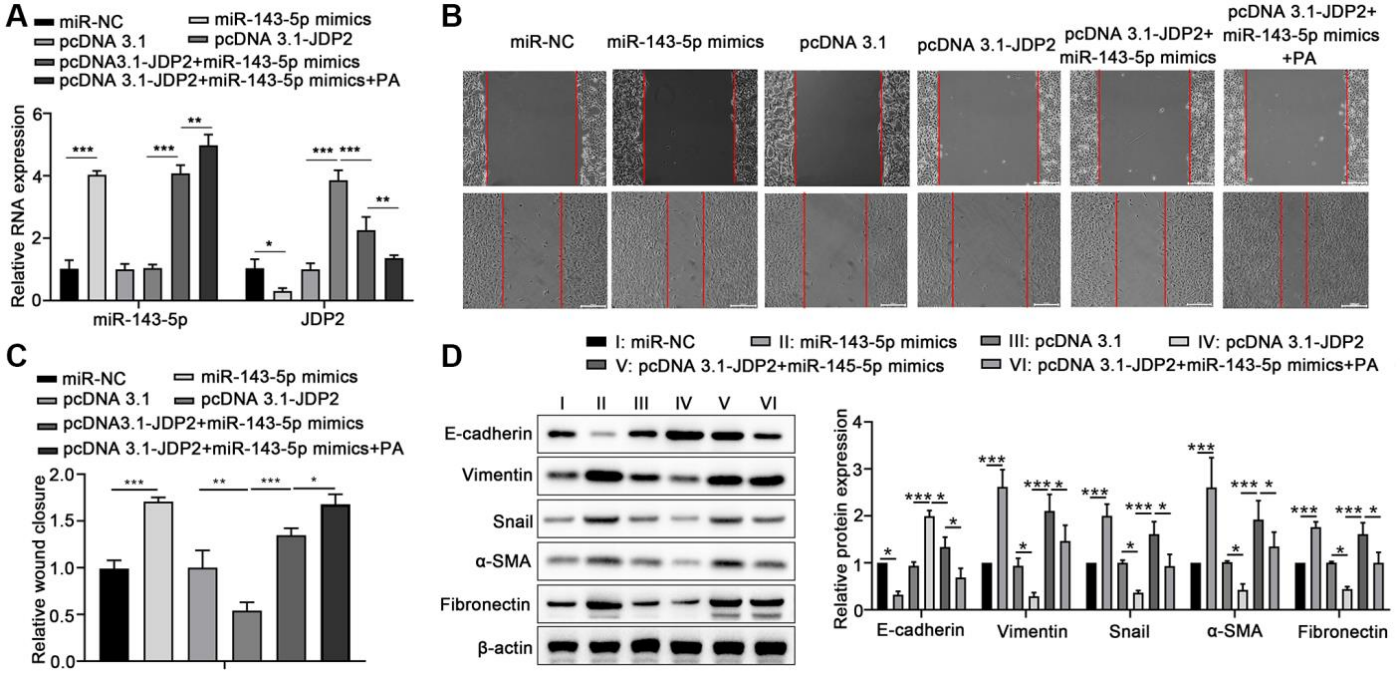
**Overexpression of JDP2 inhibited the miR-143-5p–induced EMT in ARPE-19 cells.** (**A**–**D**) ARPE-19 cells were transfected with miR-NC alone, miR-143-5p mimics alone, pcDNA 3.1 plasmid alone, pcDNA 3.1-JDP2 plasmid alone, pcDNA 3.1-JDP2 plasmid together with miR-143-5p mimics, or pcDNA 3.1-JDP2 plasmid together with miR-143-5p mimics and followed by PA treatment. (**A**) Relative levels of miR-143-5p and JDP2 mRNA in ARPE-19 cells in different treatment groups were determined via quantitative PCR. (**B**, **C**) Migration of ARPE-19 cells in different treatment groups was determined via wound healing assay. (**D**) Western blot assays determined the protein expression of E-cadherin, Vimentin, Snail, α-SMA, and Fibronectin in ARPE-19 cells in different treatment groups (*n* = 3 for each group). ^*^P < 0.05, ^**^P < 0.01, and ^***^P < 0.001, as compared with the indicated group.

Human ARPE-19 cells were transfected with miR-143-5p mimics or/and JDP2-expressing plasmid followed by PA treatment to further elucidate the underlying mechanism miRNA-143-5p/JDP2 axis in PA-induced EMT. RT-qPCR revealed that the overexpression of miR-143-5p significantly increased the expression of miR-143-5p, whereas the overexpression of JDP2 had no effect on miR-143-5p expression. However, PA treatment significantly increased the miR-143-5p expression even after miR-143-5p and JDP2 overexpression (Figure 5A). Additionally, the overexpression of miR-143-5p mimics markedly inhibited the upregulation of JDP2 after JDP2 overexpression, and PA treatment significantly enhanced the inhibitive effect (Figure 5A). Wound healing analysis revealed that miR-143-5p markedly promoted the migration of ARPE-19 cells, whereas JDP2 significantly inhibited this effect (Figure 5B, 5C). Nevertheless, the overexpression of miR-143-5p remarkably reversed the JDP2-mediated inhibition on the migration of ARPE-19 cells, whereas additional PA treatment further reversed this effect (Figure 5B, 5C). Additionally, the Western blot analysis showed that the overexpression of miR-143-5p mimics significantly decreased the E-cadherin and JDP2 expression, but increased those of Vimentin, Snail, α-SMA, and Fibronectin expression in ARPE-19 cells after JDP2 expression. Moreover, PA treatment markedly enhanced the effects of miR-143-5p mimics (Figure 5D). These data suggest that PA mediated the EMT of ARPE-19 cells via regulating the miR-143-5p/JDP2 axis.

## DISCUSSION

PA is a saturated fatty acid derived from plants that can cause aberrant miRNA expression in human cell lines [14, 15]. Recent studies have shown that PA can induce changes in cellular behaviors, including migration, proliferation, and apoptosis [32]. Hence, we hypothesized that PA-induced miRNAs are altered to promote the EMT of RPE cells. Our results revealed that miRNA-143-5p expression was increased dramatically during PA induction, and on the basis of these results, we conclude that miRNA-143-5p is involved in the EMT of RPE cells.

EMT is a prominent pathologic mechanism in ocular tissues, including cellular transdifferentiation among the lens, retina, and cornea, resulting in reduced visual axis integrity or clarity [33]. Previous studies have demonstrated that, especially in the lens and retinal tissue, the transdifferentiation of epithelial cells is one cause of fibrotic lesions [34]. EMT is a biological process in which a non-motile epithelial cell changes to a mesenchymal phenotype with invasive capacities [35]. The hallmark of EMT is the loss of epithelial surface markers, most notably E-cadherin, and the acquisition of mesenchymal markers including Vimentin, N-cadherin, and Fibronectin [35]. The transcriptional factor Snail can bind to E-boxes present in the E-cadherin promoter and transcriptionally repress the expression of E-cadherin. In addition, α-Smooth muscle actin (α-SMA) is also a frequently assessed marker in EMT, as expression of α-SMA is characteristic for myofibroblasts which are laying down an excessive amount of extracellular matrix and thus are responsible for tissue remodeling and fibrosis [36]. In this study, transfection of miR-143-5p mimics in ARPE-19 cells resulted in significantly decreased E-cadherin expression and markedly increased expression of Fibronectin, α-SMA, Snail, and Vimentin, indicating the ability of miR-143-5p in inducing EMT in human RPE cells. miRNAs are small (approximately 22 nucleotides) genetic segments that play an important role in the posttranscriptional regulation of mRNAs for translation, and exert influence on diverse biological activities [37]. For example, Yu discovered that miR-501 promoted cell invasion and EMT in hepatocellular carcinoma cells through targeting JDP2 [38]. Previous studies have shown long noncoding or circular RNAs to be miRNA-143-5p sponges that regulate EMT in different cells and tissues [39–41]. Nevertheless, we are the first to propose that miR-143-5p is directly associated with the EMT of RPE cells, which is the novelty of this study. Our discovery is consistent with the findings of Yu, in that miRNAs target JDP2 to regulate EMT [38].

miRNA-143-5p has been implicated in the pathophysiology of cancer (including colon and pancreatic cancers) and schizophrenia-related disorders [42, 43], and its effects on various target genes, such as EMC2, VWC2L, THY1, SGCZ, HIF1, and JDP2, among others, have been described. Specifically, JDP2 belongs to the basic leucine zipper transcription factor family and functions as a repressor of the AP-1 complex by dimerizing with other c-Jun proteins. Previous studies documented that JDP2 acts as a tumor suppressor in hepatocellular carcinoma and pancreatic cancer [44, 45]. We discovered that JDP2 expression was significantly downregulated after PA treatment, and JDP2 overexpression inhibited EMT in the RPE cells, a result consistent with the findings of Xu. Liu and Du discovered that overexpressed JDP2 inhibits the EMT in pancreatic cancer BxPC3 cells [46]. Wang et al. demonstrated that JDP2/Glis1/Nanog-Sall4 signaling pathway plays a key role in the induction of pluripotent stem cells from mouse embryonic fibroblasts, suggesting that JDP2 is involved in the functional transition of mouse embryonic fibroblasts [47]. In the present study, we also verified that JDP2 was a target gene of miR-143-5p and it is negatively regulated by miR-143-5p in ARPE-19 cells. Moreover, miR-143-5p overexpression could restore the EMT reduction induced by JDP2 in ARPE-19 cells, and additional PA treatment could significantly enhance this restoration, suggesting that PA promotes the development of PVR via enhancing miR-143-5p and inhibiting its regulation of JDP2 expression. Thus, JDP2 and miR-143-5p are most likely involved in the EMT of RPE cells, but no studies have elucidated the exact mechanism.

Some limitations of our research should be acknowledged. We analyzed the functional correlation between JDP2 and miR-143-5p only *in vitro* using the ARPE-19 cells, but not *in vivo*, such as in animal models. Thus, further research is necessary to confirm the functional correlation between JDP2 and miR-143-5p in ocular tissue *in vivo*. At the technical level, the incomplete blocking or excessive secondary antibody incubation time might contribute to non-specific bands on some membranes in Western blot assays. Based on previous conclusions, we plan to design related experiments to elaborate on the details of the functional correlation between JDP2 and miR-143-5p both *in vitro* and *in vivo*. In addition, since microRNAs were reported to inhibit the translation of target mRNAs [48], whether miR-43-5p can directly suppress the translation of JDP2 remains to be explored. Furthermore, since proliferation and migration can both contribute to wound closure [49], our current wound healing assay can only reflect the EMT degree of ARPE-19 cells to some extent, but was not able to distinguish the contribution of proliferation versus migration during wound closure.

In summary, our study demonstrated the stimulative effect of miR-143-5p on the PA-induced EMT of RPE cells, which is mediated via the downregulation of JDP2 expression. These results in elucidating the role of miR-143-5p and JDP2 in the EMT of RPE cells provide critical insights into the development of new therapeutics for treating PVR-related diseases.

## Supplementary Materials

Supplementary Figure 1

Supplementary Tables
