# Supplementary material for: Palmitic acid–induced microRNA-143-5p expression promotes the epithelial–mesenchymal transition of retinal pigment epithelium via negatively regulating JDP2
Source: Aging (Albany NY). 2023 Apr 25;15(9):3465–79. doi: 10.18632/aging.204684 (PMC10449279; doi:10.18632/aging.204684)
Supplement: Supplementary Figure 1 [file aging-15-204684-s001.pdf]

SUPPLEMENTARY FIGURE

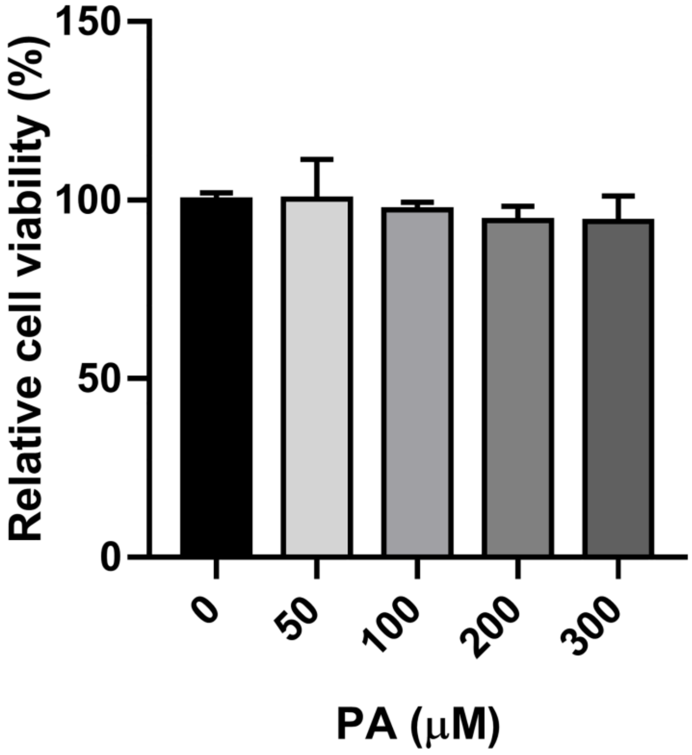

Supplementary Figure 1. PA at 0-300 μM did not decrease the viability of ARPE-19 cells. ARPE-19 cells were treated with PA at the indicated concentrations for 48 hours, and cell viability was evaluated by MTT assays.
