# Supplementary material for: Palmitic acid–induced microRNA-143-5p expression promotes the epithelial–mesenchymal transition of retinal pigment epithelium via negatively regulating JDP2
Source: Aging (Albany NY). 2023 Apr 25;15(9):3465–79. doi: 10.18632/aging.204684 (PMC10449279; doi:10.18632/aging.204684)
Supplement: Supplementary Tables [file aging-15-204684-s002.pdf]

SUPPLEMENTARY TABLES

Supplementary Table 1. Quantitative PCR primer information.

Name	Primer sequence
JDP2 forward primer	5′-CGGGGGACTGCATGGAAC-3′
JDP2 reverse primer	5′-CCAGGCATCATAGCAGGAGG-3′
E-cadherin forward primer	5′-TGGTTCAAGCTGCTGACCTT-3′
E-cadherin reverse primer	5′-CTGACCCTTGTACGTGGTGG-3′
α-SMA forward primer	5′-CCACTGCTGCTTCCTCTTC-3′
α-SMA reverse primer	5′-CGCCGACTCCATTCCAAT-3′
Snail forward primer	5′-CCAGTGCCTCGACCACTATG-3′
Snail reverse primer	5′-CTGCTGGAAGGTAAACTCTGGA-3′
Vimentin forward primer	5′-GGACCAGCTAACCAACGACA-3′
Vimentin reverse primer	5′-TCCTCCTGCAATTTCTCCCG-3′
GAPDH forward primer	5′-AATGGGCAGCCGTTAGGAAA-3′
GAPDH reverse primer	5′-GCGCCCAATACGACCAAATC-3′
miR-143-5p forward primer	5′-GCGCAGCGCCCTGTCTCC-3′
miR-143-5p reverse primer	5′-GCTGCAGAACAACTTCTC-3′
U6 forward primer	5′-GTGCTCGCTTCGGCAGCAC-3′
U6 reverse primer	5′-AAATATGGAACGCTTCAC-3′
fibronectin forward primer	5′-TCTGTGCCTCCTATCTATGTGC-3′
fibronectin reverse primer	5′-GAGGGACCACGACAACTCTTC-3′

Supplementary Table 2. Antibodies used in the study.

Antibody	Brand	Cat. No.	Working concentration
E-cadherin	Abcam	ab40772	1:30,000 (monoclonal)
α-SMA	CST	19245	1:1000 (monoclonal)
Fibronectin	Abcam	ab268020	1:1000 (monoclonal)
Snail	Abcam	ab216347	1:1000 (monoclonal)
Vimentin	Abcam	ab92547	1:2000 (monoclonal)
JDP2	Abcam	ab40916	1 μg/mL
β-actin	Abcam	ab8226	1 μg/mL
